# Highly Sensitive Oxytetracycline Detection Using QCM and Molecularly Imprinted Polymers with Deep Eutectic Solvents

**DOI:** 10.3390/polym17070946

**Published:** 2025-03-31

**Authors:** Cheng Chen, Liling Wang, Lin Xu, Houjun Wang, Peng Ye, Shuang Liao, Feng Tan

**Affiliations:** 1 School of Automation Engineering, University of Electronic Science and Technology of China (UESTC), No. 2006, Xiyuan Ave., West Hi-Tech Zone, Chengdu 611731, China; 18080696489@163.com (C.C.); 17808358208@163.com (L.W.); xulin_zoe@163.com (L.X.); 2 Shenzhen Institute for Advanced Study, University of Electronic Science and Technology of China, Shenzhen 518110, China; hjwang@uestc.edu.cn (H.W.); yepeng_uestc@163.com (P.Y.)

**Keywords:** molecularly imprinted polymer, deep eutectic solvents, quartz crystal microbalance, biosensors, antibiotics

## Abstract

This study presents an efficient method for detecting oxytetracycline, which is critical in environmental monitoring and food safety. A highly sensitive detection platform was developed by combining molecularly imprinted polymers (MIPs) with silica as a carrier, modified with deep eutectic solvents (DES), and a quartz crystal microbalance (QCM) sensor. The MIPs were specifically designed to target oxytetracycline hydrochloride, using SiO_2_ as the carrier, DES as the functional monomer, N, N-methylenebisacrylamide as the crosslinker, and ammonium persulfate as the initiator. The MIPs exhibited an adsorption capacity of 27.23 mg/g for oxytetracycline hydrochloride. After modification of the MIPs onto a gold electrode surface, a QCM-based sensor platform was constructed. The sensor demonstrated an exceptionally low detection limit of 0.019 ng/mL for oxytetracycline and exhibited excellent sensitivity in tap water. Furthermore, the sensor maintained over 90% detection performance after two weeks of room-temperature storage, indicating its stability. This method provides a rapid, highly sensitive approach for oxytetracycline detection, with potential for future improvements and widespread application in antibiotic testing.

## 1. Introduction

Oxytetracycline (OTC), a key antibiotic in the tetracycline class, is widely used in both medicine and veterinary practice [1]. However, its widespread use has raised several concerns. Animal residues may enter the human food chain, posing potential health risks. Prolonged or excessive consumption of food containing oxytetracycline hydrochloride residues may disrupt the balance of intestinal microbiota in humans, adversely affecting normal physiological functions [2,3,4]. Furthermore, these residues may cause allergic reactions, development of drug resistance, and harm the ecological environment by disrupting soil microbial communities and potentially affecting aquatic ecosystems [5,6,7,8]. Therefore, it is crucial to develop rapid, accurate, and sensitive methods for detecting oxytetracycline hydrochloride residues to monitor environmental levels and ensure both human health and ecological safety. Several routine methods have been developed for the detection of OTC, including high-performance liquid chromatography (HPLC) [9,10,11], colorimetric analysis [12,13,14,15], and liquid chromatography–tandem mass spectrometry (LC-MS) [9,16,17]. Conventional chromatography methods, such as gas chromatography-mass spectrometry (GC-MS), require laborious derivatization steps, are difficult to maintain, and suffer from low sensitivity. High-performance liquid chromatography (HPLC), while effective, is expensive, complex in terms of sample preparation, and slow in analysis. Enzyme-linked immunosorbent assay (ELISA), an immunoassay method, is susceptible to interference from antibodies and samples, leading to imprecise quantification. Fluorescence analysis also faces challenges, such as fluorescence interference, demanding handling requirements, and a limited linear detection range. These limitations hinder the fast and accurate detection of OTC, highlighting the urgent need for new, more efficient detection methods.

The Quartz Crystal Microbalance (QCM) sensor operates based on the piezoelectric effect to detect micro-masses at the nanogram (ng) level. It offers several advantages, including high sensitivity, low cost, and real-time output, positioning it as one of the most promising areas of research in sensor technology [18,19,20]. Molecularly imprinted polymers (MIP) have a highly specific recognition capability that can be tailored to bind precisely and selectively to target molecules [18,21,22]. When molecular imprinting technology (MIT) is combined with Quartz Crystal Microbalance (QCM), the advantages of both technologies complement each other. On the one hand, the specific adsorption of molecularly imprinted polymers (MIPs) enriches the target molecules on the surface of QCM, while the high sensitivity of QCM to mass changes enables the detection of trace substances with high sensitivity.

On the other hand, the selectivity of MIP compensates for QCM’s susceptibility to interference in complex samples, allowing for accurate identification of the target substances [23,24]. In practical applications, this combined technology can be used in food safety testing for rapid screening of food toxins and accurate monitoring of drug residues [25,26] in environmental monitoring for effective detection of water and air pollutants [27,28,29]; and in the biomedical field for biomarker detection and drug discovery [30,31,32]. Compared with a single technology, MIP combined with QCM significantly improves the sensitivity, selectivity, and application range.

Deep eutectic solvents (DES) are widely used in material synthesis and metal electrochemical deposition due to their low cost, environmental friendliness, biocompatibility, and biodegradability [33,34]. Their good solubility helps disperse sensitive materials, improving target material recognition. Low volatility ensures long-term sensor stability, while high ionic conductivity accelerates charge transfer, enabling rapid and sensitive sensor responses. These properties make them ideal for MIP-based QCM sensors.

SiO_2_ is often used as an ideal molecular imprinting carrier in fields such as separation, sensing, catalysis, and drug delivery [35,36]. As a molecular imprinting carrier, it features a high specific surface area, excellent mechanical strength, and chemical stability. Its surface can be easily modified, and it demonstrates good biocompatibility, making it suitable for a wide range of applications. The controllable pore structure and thermal stability further enhance its molecular recognition capabilities. Additionally, the availability of raw materials and its low cost make it ideal for large-scale applications.

**Scheme 1:**
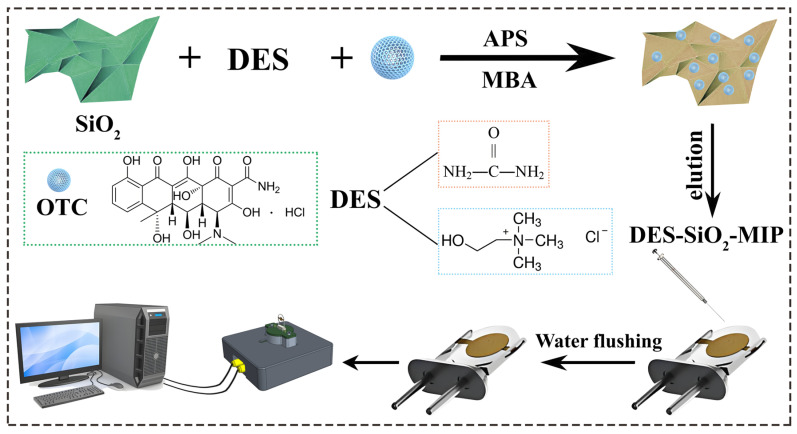
DES-SiO_2_-MIP-QCM sensor construction and OTC detection.

In this experiment, choline chloride and urea form a deep eutectic solvent (DES) that lowers the melting point, facilitating polymerization. SiO_2_ serves as the stabilizing carrier, while OTC acts as the template molecule, and the DES creates a functional environment for molecular imprinting [37]. N, N-methylenebisacrylamide (MBA) is used as the crosslinker, and ammonium persulfate (APS) initiates the polymerization process. OTC forms a complex with the DES on the surface of SiO_2_ through hydrogen bonding, electrostatic interactions, and other forces. APS decomposes to generate free radicals, initiating the polymerization of DES and MBA. The double bonds of MBA react with active groups in the DES, forming a crosslinked network in which OTC is encapsulated. After washing with a methanol and acetic acid mixture, OTC is removed, leaving behind imprinted cavities that are complementary in shape and functional groups to OTC. Urea in the DES interacts with OTC’s amino and hydroxyl groups, enhancing selectivity [38,39]. The crosslinker ensures a stable 3D polymer network, creating specific recognition sites for OTC, enabling effective and selective detection of OTC hydrochloride (Scheme 1).

## 2. Materials and Methods

### 2.1. Reagents and Materials

The compounds used in this study, including oxytetracycline (OTC) (CAS: 79-57-2), tetracycline (TTC) (CAS number: 60-54-8), doxycycline (DOX) (CAS: 564-25-0), chlortetracycline (CTC) (CAS: 57-62-5), SiO_2_ (CAS: 60676-86-0), choline chloride (CAS: 67-48-1), urea (CAS: 57-13-6), N, N-methylenebis acrylamide (CAS: 110-26-9), ammonium persulfate (CAS: 7727-54-0), acetic acid (CAS: 64-19-7), and methanol (CAS: 67-56-1), were sourced from McLean Biochemicals (Shanghai, China) Co. All reagents were of analytical reagent grade for high purity. Deionized water (resistivity 18.25 MΩ·cm) was used throughout to maintain experimental integrity and avoid ion or contaminant interference.

To prepare the stock solution of OTC, dissolve 5.0 mg of OTC in 100.0 mL of methanol, resulting in a final concentration of 50.0 µg/mL. Store the stock solution in a dark container to protect it from light. For the standard working solution, dilute the stock solution with deionized water to the desired concentration, ensuring proper preparation for subsequent experiments.

### 2.2. Instrumentation

Scanning electron microscopy (SEM) was used to analyze the generated samples’ appearance and structure (Sigma 500, ZEISS, Oberkochen, Germany). To determine elemental compositions and valence states, X-ray photoelectron spectroscopy (XPS) (ESCA-LAB-250Xi, Thermo Fisher Scientific, Waltham, MA, USA) was employed. X-ray diffraction (XRD) (DX-2700, Scan range: 5–80°, Scan Speed: 5°/min, Rigaku, Dandong, China) was utilized to characterize the crystalline structure. Fourier transform infrared spectroscopy (FTIR) measurements, conducted with attenuated total reflection (ATR) (IS50, Spectral Resolution: 0.25 cm^−1^, Thermo Fisher Scientific, Waltham, MA, USA), provided additional structural insights. The UV–visible absorption spectra of the OTC supernatant were acquired using a UV–visible spectrophotometer (UV755B, Wavelength Range: 190 nm~1100 nm, Spectral Bandwidth: 2 nm, Yoke, Shanghai, China). Contact angle measurements were performed using the stationary drop method with a KRÜSS DSA100 (Hamburg, Germany). The surface morphology of both QCM and MIP-QCM chips was observed using an atomic force microscope (AFM) (Bruker, Billerica, MA, USA). AT-cut 10 MHz quartz crystals (8 mm in diameter) with gold electrodes (3 mm in diameter) were obtained from Chengdu Tian’ao Electronics Company Limited (Chengdu, China). Sensor parameters were evaluated with Saunders & Associates (250B-2, Saunders, Englewood, CO, USA).

### 2.3. Preparation of Molecularly Imprinted Polymers with Deep Eutectic Solvents

#### 2.3.1. Preparation of Deep Eutectic Solvents

Choline chloride and urea, in a 1:2 molar ratio, were heated and stirred in a round-bottom flask at 80 °C for 30 min in a water bath. The mixture gradually transitioned from solid to a homogeneous, transparent liquid, confirming successful preparation of the deep eutectic solvent.

#### 2.3.2. Preparation of DES-SiO_2_-MIP

To prepare the MIP, dissolve 100 mg of SiO_2_ in 30 mL of PBS buffer containing 20 mg of OTC and 100 µL of DES solution. Deoxygenate the mixture by passing nitrogen through it for 15 min to remove dissolved oxygen. Subsequently, add 100 mg of N, N-methylenebis acrylamide (MBA) and 10 mg of ammonium persulfate (APS) to the solution, and stir the mixture at 50 °C for 24 h. After the reaction, centrifuge the mixture to isolate the uneluted MIP. The resulting MIP can then be further characterized or applied as needed.

Upon completion of polymerization, the polymers were eluted several times with a methanol-acetic acid (9:1, *v*/*v*) mixture to ensure complete removal of the template. After the elution process, the polymer was thoroughly washed with methanol and ultrapure water to eliminate any remaining solvents and impurities. To create DES-SiO_2_-MIP, the cleaned polymer was subsequently vacuum-dried for 24 h at 60 °C.

### 2.4. Adsorption Performance Study of DES-SiO_2_-MIP

Eluting agent selection: The materials were divided into three parallel groups and eluted with four different eluents: (1) 100% methanol, (2) 90% methanol + 10% acetic acid, (3) 100% ethanol, and (4) 10% methanol + 90% water. The eluents were applied under identical conditions for the same duration. UV spectrophotometric analysis at 353 nm was used to evaluate the elution efficiency.

Optimization of adsorption temperature and time was conducted by varying the reaction temperature (20 °C, 30 °C, 40 °C, and 50 °C) and adsorption time (20, 40, 60, 80, 100, and 120 min), while keeping all other conditions constant. After the adsorption process, the supernatant was centrifuged, and its absorbance was measured at 353 nm to assess the effectiveness of the adsorption under different conditions. The optimal conditions were determined based on the absorbance data obtained.

Take the OTC standard solution and dilute it to different concentrations (2–50 μg/mL), measure its absorbance at each concentration, and plot a standard curve for the calculation of subsequent adsorption data.

DES-SiO_2_-MIP or DES-SiO_2_-NIP (1 mg/mL, 0.2 mL) was incubated in OTC solutions of different concentrations (2–50 μg/mL, 1.8 mL). After two hours of adsorption, centrifugation (6000 rpm) was performed to separate, and the supernatant was collected to determine the OTC concentration using a UV spectrophotometer. The adsorption capacity was calculated using the following formula:

(1) $$Q_{e}=(C_{0}-C_{e})\times V/m$$

where C_0_ (μg/mL) and C_e_ (μ/mL) are the initial and final concentrations of OTC, respectively; *V* (mL) is the volume of the solution; *m* (mg) is the mass of the polymer.

The selectivity of the adsorbent is described using the imprinting factor α and the selectivity factor β as indicators. The higher the value, the stronger the specific adsorption capacity.

(2) $$\alpha =Q_{MIP}/Q_{NIP}$$

(3) $$\beta =\alpha_{T}/\alpha_{C}$$

where *Q_MIP_* is the adsorption capacity of the MIP (mg/g), *Q_NIP_* is the adsortion capacity of the NIP (mg/g), *α_T_* is the imprinting factor of the target pollutant, and *α_C_* is the imprinting factor of the competitive pollutants.

### 2.5. Preparation of DES-SiO_2_-MIP-QCM Sensor

Quartz crystal gold electrodes were thoroughly cleaned and dried. The quartz crystal gold electrodes were cleaned in ethanol by ultrasonication before modification, and then immersed in acidic piranha solution (30% H_2_O_2_/concentrated H_2_SO_4_ = 1:3, *v*/*v*) for 5 min. After thorough cleaning, the electrodes were dried with nitrogen. Next, accurately weigh 10 mg of DES-SiO_2_-MIP and dissolve it in ultrapure water to form a homogeneous suspension with a concentration of 1 mg/mL. This suspension was then spin-coated onto the surface of the electrode at room temperature. After coating, the electrodes were kept dry to ensure proper adhesion of the DES-SiO_2_-MIP layer.

The amount of electrode coating was optimized by testing 0.5, 1, 1.5, 2, 3, 4, and 5 µg of DES-SiO_2_-MIP. The effect on the QCM sensor’s response frequency was measured to determine the optimal DES-SiO_2_-MIP-QCM coating amount for the best sensor performance.

### 2.6. QCM Sensor Measurements

A dropwise addition of 1 µL of OTC standard solution at several concentrations was made to the MIPs-QCM sensor’s surface. For adsorption, the sensor was let to acclimate for ten minutes at room temperature. Following equilibration, the unbound OTC was removed using deionized water, the chip was dried, and the frequency change was measured. A frequency–concentration calibration curve was produced using the electrode frequency prior to the dropwise addition of the measured substance as a reference. By performing QCM analysis with equal doses of several antibiotic analogs added dropwise under identical conditions, the selectivity was confirmed. In order to evaluate the MIPs-QCM sensor’s reusability, it can be submerged in a solution of methanol and acetic acid to rinse the OTC analyte’s imprinted sites, elute the OTC, dry the chip, repeat the previous steps, and then adsorb it once more. This process is repeated five times, after which the sensor’s sensitivity is measured.

The calculation formulas for detection limit (LOD) and relative activity (RA) are as follows:

(4) LOD=3σ/S

where *σ* is standard deviation of the blank (often the baseline noise); *S* the is slope of the calibration curve.

(5) $$RA=F_{A}/F_{S}\times 100\%$$

where *F_A_* is actual frequency; *F_S_* refers to the frequency measured for the first time.

### 2.7. Preparation of Real Samples

A measured volume of tap water was filtered through a 0.45 μm membrane to remove suspended particulate matter. The target compounds were subsequently eluted using a suitable eluent to obtain the sample solution for analysis. The resulting sample solution was stored in a refrigerator at 4 °C to preserve its stability and was shaken thoroughly before use to ensure homogeneity.

## 3. Results and Discussion

### 3.1. Design and Preparation of DES-SiO_2_-MIP

In this study, a molecularly imprinted polymer (DES-SiO_2_-MIP) was synthesized by polymerizing MIP onto the surface of SiO_2_. During the synthesis, DES, N,N-methylenebisacrylamide (MBA), OTC, ammonium persulfate (APS), and SiO_2_ functioned as the functional monomer, crosslinking agent, template, initiator, and carrier, respectively. The novelty of this study lies in its application of DES in the preparation of MIPs. The molecularly imprinted polymer is formed through the following steps: (1) The template molecule interacts with the functional monomer through various forces, forming a complex on the surface of the carrier. (2) A crosslinking agent is added to the complex, and the initiator decomposes to generate free radicals, initiating the polymerization of DES and MBA to form a crosslinked network. During this process, the template molecule is “captured” within the stereostructure of the MIP. (3) The template is removed from the MIP using a methanol and acetic acid mixture, leaving behind binding sites that specifically recognize the imprinted molecule.

The primary role of DES is to immobilize OTC through hydrogen bonding. The nitrogen and hydrogen atoms in choline chloride and urea form hydrogen bonds with the amino, carboxyl, and other polar groups in the tetracycline molecule. Furthermore, the polymerization reaction requires a stable chemical solvent, and the low eutectic system formed by choline chloride and urea serves as an effective solvent, providing a favorable chemical environment for the reactants, including the crosslinking agent and initiator. Finally, a methanol–acetic acid (9:1, *v*/*v*) mixture is used as the eluent to remove OTC, successfully preparing the DES-SiO_2_-MIP.

### 3.2. Morphological and Structural Characterization of DES-SiO_2_-MIP

**Figure 1:**
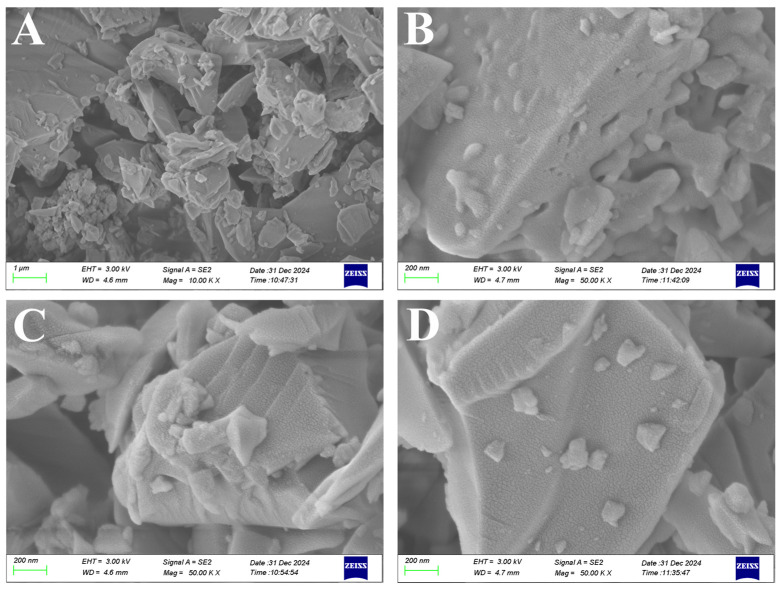
SEM images of DES-SiO_2_-MIP at different magnifications. (**A**) 10^4^ × (**B**) 5 × 10^4^ ×. (**C**) SEM images of SiO_2_. (**D**) SEM images of DES-SiO_2_-NIP.

The SEM images of DES-SiO_2_-MIP at various magnifications provide valuable insights into its hierarchical structure and surface morphology, both of which significantly influence its adsorption efficiency. Figure 1A presents irregular three-dimensional structures of considerable size, reflecting the bulk architecture of the material. This structural complexity may enhance the material’s functional properties. Figure 1B, captured at a magnification of 5 × 10^4^, reveals rough surfaces with pronounced irregularities and cavities. These features are particularly important, as they increase the active surface area, thereby improving the material’s adsorption capacity. The surface irregularities and cavities likely provide additional interaction sites for target molecules, further optimizing the material’s performance in adsorption applications.

The morphology of DES-SiO_2_-MIP (Figure 1B) and DES-SiO_2_-NIP (Figure 1D) closely resembles that of bare silica (Figure 1C). However, the surfaces of DES-SiO_2_-MIP and DES-SiO_2_-NIP are rougher than bare silica, indicating that polymerization has successfully modified the surface. Overall, the SEM analysis highlights the potential of DES-SiO_2_-MIP, with its enhanced surface area and complex morphology to achieve superior adsorption efficiency.

**Figure 2:**
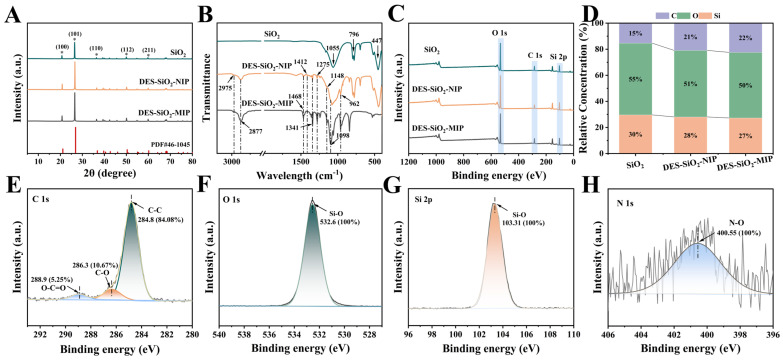
Structural characterization of materials. (**A**) XRD patterns, (**B**) FTIR spectra, (**C**) XPS total spectrum, and (**D**) the relative concentration changes of each element of SiO_2_, DES-SiO_2_-NIP, and DES-SiO_2_-MIP. (**E**) C 1s, (**F**) O 1s, (**G**) Si 2p, and (**H**) N 1s of DES-SiO_2_-MIP.

The DES-SiO_2_-MIP is further characterized in detail using X-ray diffraction (XRD), Fourier-transform infrared (FTIR) spectroscopy, and X-ray Photoelectron Spectroscopy (XPS). XRD analysis (Figure 2A) reveals that both the synthesized non-imprinted polymer (NIP) and the molecularly imprinted polymer (MIP) display distinct diffraction peaks corresponding to SiO_2_, indicating that the material retained its excellent crystalline properties. These results suggest that the integrity of the SiO_2_ structure was preserved throughout the modification process, with no significant changes to its crystalline form.

The functional groups of the various synthesize nanoparticles are analyzed using Fourier-transform infrared (FT-IR) spectroscopy. The corresponding spectra are presented in Figure 2B. In the SiO_2_ spectrum, characteristic peaks at 796 cm^−1^ and 1055 cm^−1^ confirm the presence of SiO_2_, while the bands observed between 447 cm^−1^ and 796 cm^−1^ are attributed to Si-O vibrations, further validating the silica structure [40]. For DES-SiO_2_-NIP and DES-SiO_2_-MIP, new characteristic peaks were observed at 2975 cm^−1^ (O-H stretching) and 2877 cm^−1^ (C-H stretching), indicating the presence of choline chloride (ChCl) and urea (UR) [41,42], the peaks at 1412 cm^−1^ and 1098 cm^−1^ correspond to the stretching vibrations of C–N and C–C in DES [43,44], respectively. These peaks suggest that ChCl, acting as a hydrogen bond acceptor (HBA), and urea, functioning as a hydrogen bond donor (HBD), interact strongly with each other, confirming the successful formation of the deep eutectic solvent (DES). The peak at 1143 cm^−1^ in the DES-SiO_2_-MIP spectrum shows a clear C-O-C stretching vibration, which suggests that this absorption peak may be related to the structural interaction between the crosslinker (MBA) and DES during the synthesis process [45]. The appearance of the C-O-C stretching peak further confirms the successful synthesis of the molecularly imprinted polymer. The peak at 1275 cm^−1^ may be related to the vibration of C-N or C-O bonds in the crosslinker MBA [46,47]. Additionally, the peak at 1468 cm^−1^ corresponds to C-H bending vibrations, which is characteristic of both the MIP and NIP samples, further supporting the successful synthesis of DES-SiO_2_-MIP [48]. Additionally, it is noted that there is a significant difference in peak intensity between MIP and NIP in the FTIR spectra, which is mainly due to the molecular imprinting effect, the interaction between the template molecules and the polymer, and the enhanced response of certain specific functional groups. These factors work together to make the FTIR peaks in MIP more pronounced.

The elemental composition of DES-SiO_2_-MIP was analyzed using X-ray photoelectron spectroscopy (XPS), as shown in Figure 2C. The results reveal that the primary components o the DES-SiO_2_-MIP samples are silicon, oxygen, and trace amounts of carbon. As shown in Figure 2D, the oxygen and silicon peaks of DES-SiO_2_-MIP and DES-SiO_2_-NIP are lower compared to SiO_2_. This is mainly due to the molecularly imprinted polymers covering or chemically modifying the surface of SiO_2_, which reduces the measurable SiO_2_ components on the surface and decreases the signal intensity of oxygen and silicon.

On the other hand, the carbon peak is enhanced due to the large amount of organic carbon present in the polymer. The formation of the polymer increases the surface concentration of carbon, thereby intensifying the carbon peak signal in XPS. In Figure 2E, the XPS spectrum shows that carbon is predominantly present in the forms of C-C, C-O, and O-C=O bonds [49]. Among these functional groups, oxygen atoms are negatively charged and can readily interact with hydroxyl groups in SiO_2_. This suggests that the observed carbon likely originates from organic residues left over during the material’s preparation process. The O1s spectrum (Figure 2F) displays a distinct peak, indicating the presence of oxygen atoms, primarily associated with SiO_2_ as well as hydroxyl or carboxyl groups, thereby confirming the preservation of the silica network [50]. The high-resolution Si 2p spectrum (Figure 2G) shows a prominent peak at 106.6 eV, confirming that the silicon in DES-SiO_2_-MIP remains in the form of SiO_2_, maintaining strong Si-O interactions [51,52]. Additionally, we observed that the XPS peak for nitrogen appears at 400.55 eV and is relatively weak (Figure 2H). This may be the characteristic peak of amide or amine-type nitrogen, which is consistent with the chemical structure of the crosslinking agent in the molecularly imprinted polymer. The lower peak intensity may be due to the influence of silica on the nitrogen, which interacts with the oxygen functional groups on the silica surface to form stable chemical bonds, existing in a chemical form that is not easily dissociable.

**Figure 3:**
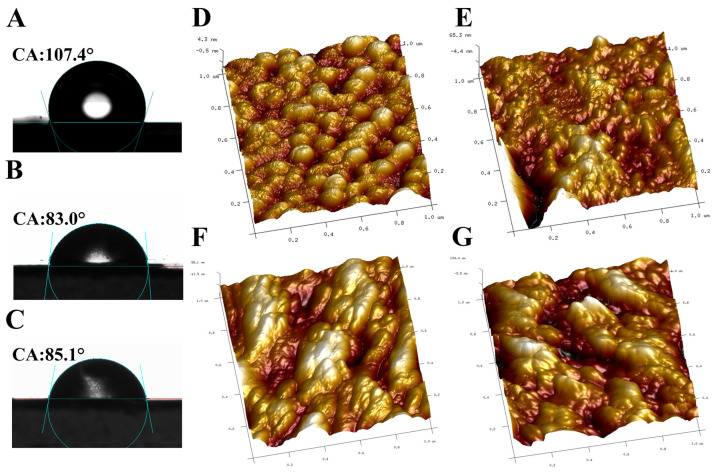
Surface Characterization of DES-SiO_2_-MIP-QCM Sensors. Contact angle of (**A**) bare chip, (**B**) DES-SiO_2_-NIP-QCM, and (**C**) DES-SiO_2_-MIP-QCM. AFM 3D images of (**D**) bare chip, (**E**) DES-SiO_2_-NIP-QCM, (**F**) DES-SiO_2_-MIP-QCM before elution, and (**G**) DES-SiO_2_-MIP-QCM.

In this study, DES-SiO_2_-MIP-QCM and DES-SiO_2_-NIP-QCM sensors are prepared by immobilizing DES-SiO_2_-MIP on the surface of QCM electrodes using the spin-coating method. The sensors are characterized by contact angle (CA) measurements and atomic force microscopy (AFM), as shown in Figure 3A–C. The unmodified QCM surface exhibit a CA value of 107.4°. For the modified surfaces, the CA values are 83.0° for NIP and 85.3° for MIP, confirming the successful attachment of the material to the chip surface and its good hydrophilicity.

AFM is also used to examine the surface morphology and roughness (Rq) of MIP before and after elution (Figure 3D–G). The Rq value of MIP before elution is 18.6 nm, while the Rq value after elution increased to 30.2 nm, approximately 1.6 times greater than before. This is because the removal of OTC from MIP left behind cavities and increased surface roughness, which in turn raised the Rq value. In contrast, NIP exhibit a denser membrane, with an Rq value of 17.6 nm (Table 1). Additionally, the polymer film thicknesses of MIP and NIP are 31.0 nm and 33.45 nm, respectively, whereas the thickness of the eluted MIP film is 29.56 nm. This reduction in thickness is likely due to the hydrophilicity of MIP, which caused slight film loss during the elution process. In conclusion, the polymer film is effectively adhered to the gold surface of each QCM sensor and demonstrate the capability to successfully elute the template molecules.

### 3.3. Investigation of the Adsorption Properties of DES-SiO_2_-MIP

**Figure 4:**
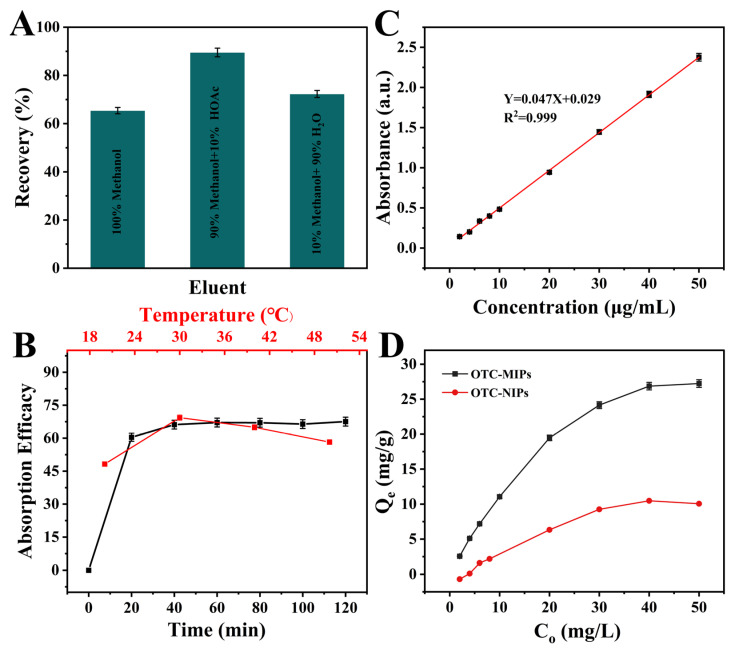
Investigation of the adsorption properties of DES-SiO_2_-MIP. (**A**) Selection of eluent. (**B**) Optimization of adsorption temperature and time. (**C**) Linear curve of OTC standard solution. (**D**) Adsorption saturation curves for DES-SiO_2_-NIP and DES-SiO_2_-MIP.

The impact of different eluents on elution efficiency is initially investigated, with a mixture of 90% methanol and 10% acetic acid identified as the optimal eluent (Figure 4A). The assay temperature plays a significant role in the adsorption capacity of OTC onto molecularly imprinted polymers. Experimental trials are conducted at various temperatures (20 °C, 30 °C, 40 °C, and 50 °C). The results show that adsorption initially increases with temperature, reaching a peak at 30 °C, and then decreases as the temperature rises further (Figure 4B). This behavior is likely due to enhanced molecular movement at higher temperatures, which promotes the diffusion of OTC molecules into the pores of the molecularly imprinted polymer, facilitating their binding to the imprinted sites. However, at excessively high temperatures, the intermolecular forces may weaken, leading to a reduction in adsorption capacity. Therefore, 30 °C is selected as the optimal detection temperature.

To optimize the adsorption time, various intervals (20, 40, 60, 80, 100, and 120 min) were tested. The concentration of template molecules remaining in the supernatant after adsorption was measured to determine the optimal time. It was observed that once the adsorption time exceeded 40 min, there was no significant change in the concentration of template molecules in the supernatant. Therefore, 40 min was chosen as the optimal adsorption time (Figure 4B).

The calibration curves are constructed using a range of OTC standard solution concentrations (2 to 20 μg/mL), resulting in a linear regression equation of Y = 0.047X + 0.029, with a correlation coefficient of R^2^ = 0.999 (Figure 4C). To optimize the detection performance, several experimental conditions, including eluent choice, adsorption temperature, and adsorption time, were evaluated.

The isothermal adsorption test was conducted under optimal conditions, as shown in Figure 4D. The results demonstrate that DES-SiO_2_-MIP exhibits nearly twice the adsorption capacity of DES-SiO_2_-NIP. Upon reaching saturation, the adsorption capacity of DES-SiO_2_-MIP is 27.23 mg/g, significantly higher than the 10.03 mg/g observed for DES-SiO_2_-NIP. To assess selectivity, three tetracycline antibiotics structurally similar to OTC, namely TTC, DOX, and CTC, were tested as potential interferents. The imprinting factors (α) and selectivity factors (β) for these interferents were compared, and the calculation results are presented in Table 2. The data indicate that the α and β values for all three similar compounds are greater than 1, suggesting that, compared to similar compounds such as DOX, CTC, and TTC, the MIP is more effective at recognizing and adsorbing OTC. This finding implies that the MIP material exhibits strong selective recognition of OTC.

**Figure 5:**
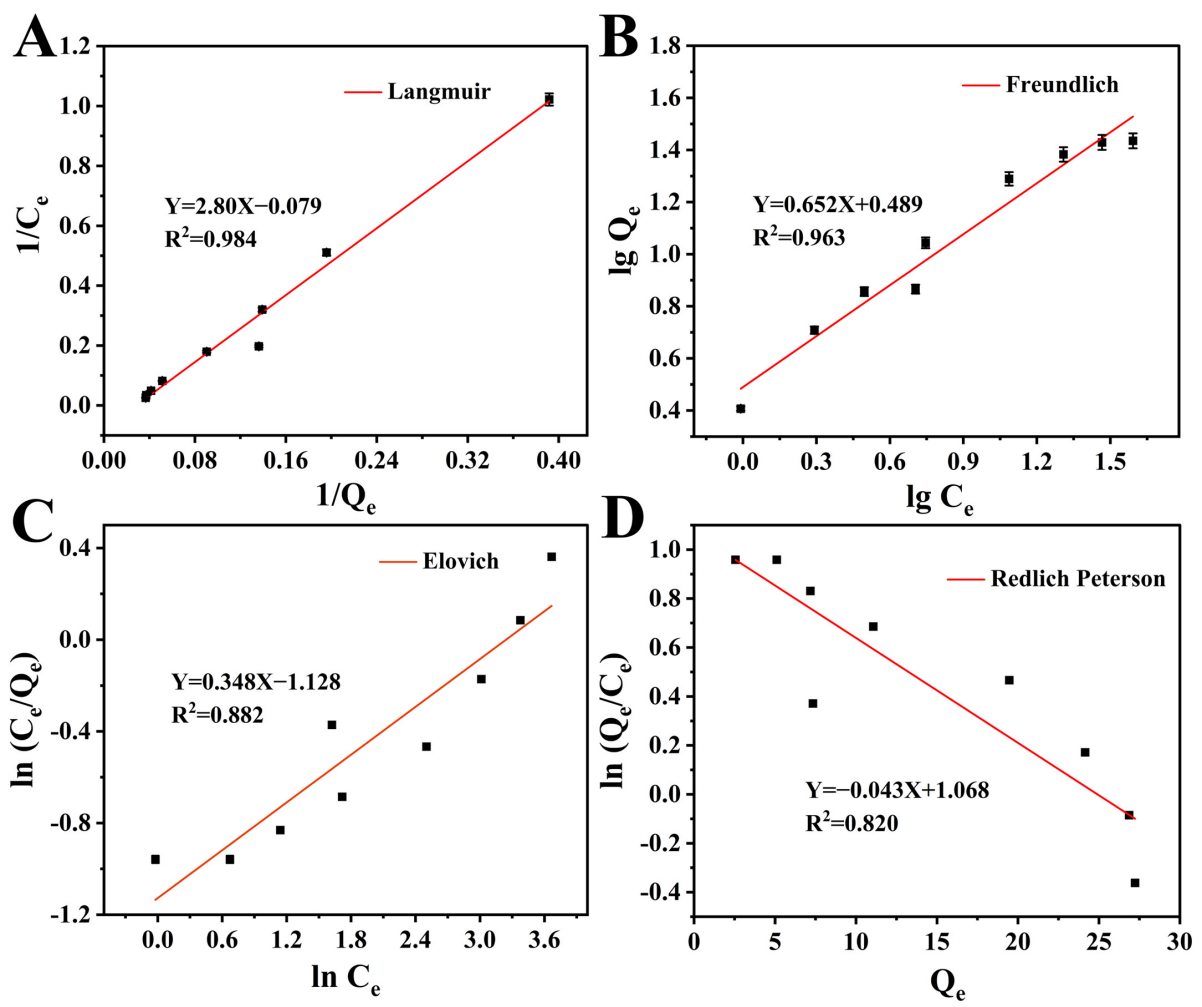
Testing fit of adsorption isotherms for the OTC imprinted sensor. (**A**) Langmuir, (**B**) Freundlich, (**C**) Elovich, and (**D**) Redlich–Peterson.

Adsorption modeling is a critical component of MIP studies, as it provides valuable insights into the homogeneity of adsorption sites and helps elucidate the nature of the adsorption process. In this study, the adsorption of MIPs to OTC is investigated, and the experimental data are analyzed using four widely recognized equilibrium isotherm models: Langmuir, Freundlich, Elovich, and Redlich–Peterson (Figure 5), for which their linearized equations are depicted as follows:

(6) Langmuir: 1Q=1bQmax×1C×1Qmax

(7) Freundlich: ln(Q)=1nlnC+ln(Qmax)

(8) Redlich–Peterson: ln(CQ)=βlnC−lnA

(9) Elovich: ln(QC)=−1Qmax×Q+ln(KeQmax)

where *Q* and *Q_max_* are the equilibrium and maximum binding capacity (mg/g) and correlated with the mass loading (Δ*m*) per unit area of QCM sensor for a given TCF concentration calculated from the Δ*F* measured, *C* refers to the concentration (mg/L) of TCF at equilibrium, b refers to the Langmuir constant, nF a Freundlich exponent which provides an indication of adsorption intensity, *β* refers to a Redlich–Peterson exponent, *A* refers to the Redlich–Peterson constant and *K_e_* refers to the Elovich constant.

The linearized forms of these isotherms are commonly employed to interpret the adsorption data and evaluate the model’s fit to the experimental results [53]. The correlation coefficient (R^2^) is used to evaluate the fit of the isotherms to the experimental data obtained at equilibrium. The Langmuir isotherm displayed the highest correlation coefficient (R^2^ = 0.984), indicating the best fit among the four models (Figure 5A). This suggests that the adsorption process may involve simultaneous interactions exhibiting Langmuir characteristics, implying that the adsorption of DES-SiO_2_-MIP follows a homogeneous adsorption mechanism.

### 3.4. DES-SiO_2_-MIP-QCM Detection

**Figure 6:**
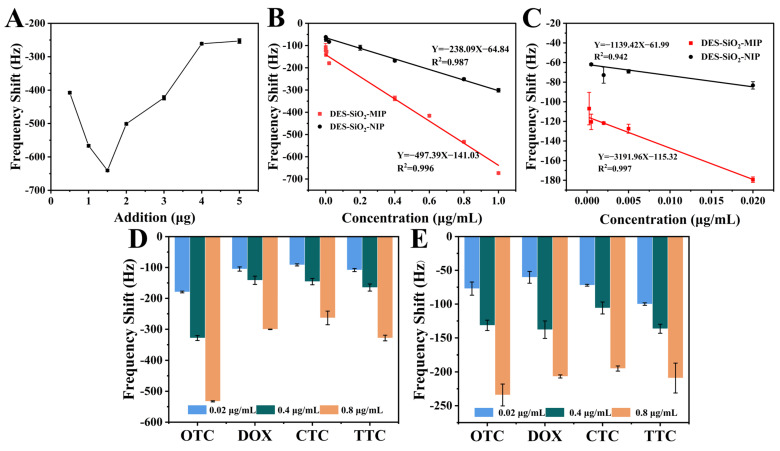
Detection performance of DES-SiO_2_-MIP-QCM sensors. (**A**) Frequency shift of the sensor with the different additions of DES-SiO_2_-MIP. (**B**) Linear curve of DES-SiO_2_-NIP-QCM and DES-SiO_2_-MIP-QCM for OTC detection. (**C**) A partially enlarged view of the linear curve in (**B**). (**D**) The selective adsorption of DES-SiO-MIP-QCM (at three concentrations). (**E**) The selective adsorption of DES-SiO_2_-NIP-QCM (at three concentrations).

The number of imprinted binding sites on the modified membrane is a critical factor influencing the sensor’s sensitivity and response time. As shown in Figure 6A, the sensor’s frequency shift increases as the MIP coating amount rises from 0.5 to 1.5 µg, reaching an optimal peak at 1.5 µg. This suggests that, at this coating level, the sensor achieves the optimal balance between the number of imprinted sites and adsorption capacity, resulting in the highest sensitivity and fastest response time. However, when the MIP coating exceeds 1.5 µg, the frequency shift begins to decline. This observation indicates that an excessive MIP coating may cause overcrowding of binding sites, potentially impairing selective analyte adsorption. Consequently, sensor performance deteriorates, likely due to reduced binding site accessibility or structural hindrance, which interferes with the sensor’s ability to effectively interact with and detect the target analyte. Therefore, optimizing the MIP content is crucial to achieving the best performance in terms of sensitivity, selectivity, and response time.

In this study, various concentrations of OTC (ranging from 0.00025 to 1 μg/mL) were tested using an DES-SiO_2_-MIP-QCM sensor under optimized conditions (Figure 6B). The results demonstrate a strong linear correlation between OTC concentration and the corresponding frequency shifts, indicating that the target analyte can be accurately detected and quantified by the sensor within this concentration range. A similar linear trend is observed with the DES-SiO_2_-NIP-QCM (Non-Imprinted Polymer QCM), which follows the same general equation for the frequency–concentration relationship. However, the DES-SiO_2_-MIP-QCM exhibits more than twice the sensitivity of the NIP-QCM, highlighting that molecular imprinting significantly enhances the sensor’s performance. This improvement is attributed to the selective binding sites formed in the MIP, which enable more specific interactions with the target analyte compared to the NIP. Figure 6C presents an enlarged view of the linear curve for concentrations below 0.02 μg/mL. The linear slope in the low concentration region is significantly higher than that in the high concentration region. The main reason for this phenomenon is that when OTC molecules interact with the DES-SiO_2_-MIP-QCM sensor surface, they exhibit a higher specificity in adsorption, which results in higher sensor sensitivity in the lower concentration range. However, as the OTC concentration increases, the spatial limitations of the imprinted cavity lead to a significant decrease in the sensor’s response slope. This phenomenon may be related to the competitive effect of molecules within the imprinted cavity, which slows down the adsorption rate and thus affects the slope of the linear fitting curve at higher concentrations.

In summary, the sensor has two different linear ranges: 0.00025~0.02 μg/mL and 0.02~1 μg/mL. The change in slope at 0.02 μg/mL may be due to adsorption kinetics. Based on the low concentration linear range obtained low detection limit (LOD) of 0.019 ng/mL. This demonstrates the high sensitivity of the MIP-based sensor, making it an effective tool for the accurate quantification of trace-level analytes, such as OTC, in various samples. The enhanced sensitivity and selectivity of the DES-SiO_2_-MIP-QCM system position it as a promising candidate for precise and sensitive analytical applications.

To evaluate the selectivity, three tetracycline antibiotics with structures similar to OTC were tested as potential interferents (Figure 6D,E). Each interferent was tested at three different concentrations. The results indicated that the response of DES-SiO_2_-MIP to OTC was significantly higher than that to the analogs at all concentrations. In contrast, DES-SiO_2_-NIP showed minimal difference in the detection of these four substances, suggesting that the DES-SiO_2_-MIP exhibits strong selectivity for OTC. The corresponding selectivity factors were subsequently calculated to more effectively highlight the distinctions between DES-SiO_2_-MIP and DES-SiO_2_-NIP, further reinforcing the superior selectivity of MIP (Table 3).

**Figure 7:**
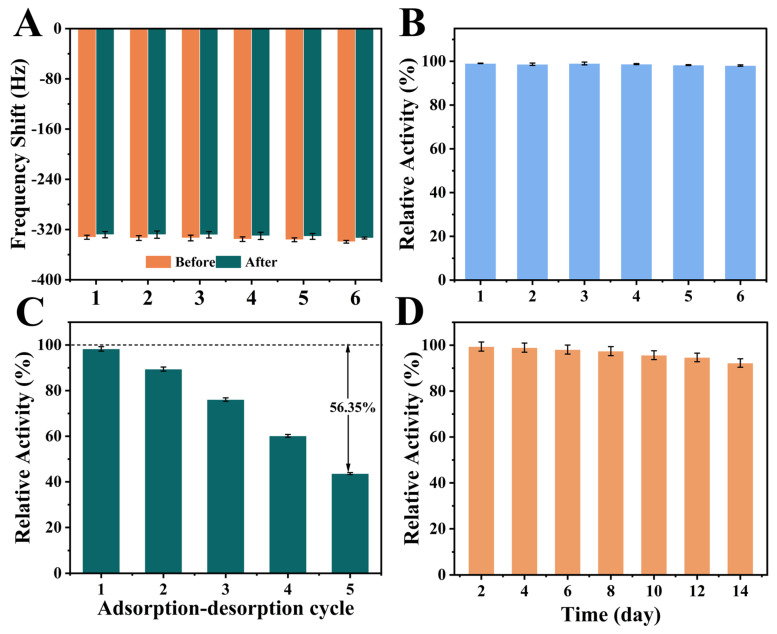
(**A**) The frequency changes in the adsorption-elution-re-adsorption experiments of six parallel sensors. (**B**) The relative activity corresponding to A. (**C**) Elution stability and (**D**) temporal stability of the DES-SiO_2_-MIP-QCM.

To assess reproducibility, sensors from the same batch of chips were used. Six sensors were randomly selected from this batch for a single adsorption-elution-re-adsorption cycle experiment, while DES-SiO_2_-MIP-QCM performance tests were conducted simultaneously. The frequency values of the different chips were recorded and compared, and their relative activity values were calculated to evaluate the consistency and variability of the batch’s performance. The results shown in Figure 7A,B demonstrate that the relative activity values exceed 98%, confirming the exceptional stability and reliability of the DES-SiO_2_-MIP-QCM sensors. This ensures consistent sensor performance across tests and provides a reliable foundation for monitoring, preventing significant data deviations due to sensor variations.

The stability of DES-SiO_2_-MIP-QCM was evaluated from two key aspects: elution stability and time stability. Regarding elution stability (Figure 7C), the effect of multiple adsorption-elution-re-adsorption cycles on DES-SiO_2_-MIP-QCM performance was examined in detail. As the number of elution cycles increased, a gradual decrease in the binding ability between MIP and the QCM surface was observed. After five cycles, the relative activity dropped below 50%. This decline in binding ability is primarily due to interactions between the eluent and MIP during the elution process, which gradually disrupts the bond between MIP and QCM. It may also lead to degradation or detachment of some MIP structures. This change is visually represented in the mass change, which decreases progressively as the number of cycles increases. These findings indicate that DES-SiO_2_-MIP-QCM response performance deteriorates with an increasing number of elution cycles, suggesting that elution stability requires improvement.

From the perspective of time stability (Figure 7D), the performance changes of DES-SiO_2_-MIP-QCM after long-term storage at room temperature were investigated. The experimental results show that, even after two weeks of storage at room temperature, the relative activity of DES-SiO_2_-MIP-QCM remains above 90%. This high relative activity indicates that, despite long-term storage, the adsorption and detection capabilities of DES-SiO_2_-MIP-QCM for the target substance remain at a high level, strongly confirming the sensor’s excellent time stability.

In addition, the analytical performance of the current study is compared with that of previous OTC assays, as summarized in Table 4. The results indicate that the detection limit achieved in this study is lower than that of most previously reported methods. Furthermore, the present method offers a wide linear range and does not require complex chemical modifications, emphasizing its simplicity and sensitivity compared to other approaches.

### 3.5. Actual Sample Analysis

The OTC content of the pre-treated water samples was measured directly, it is observed that no OTC was detected in the untreated tap water sample. Subsequently, a more comprehensive evaluation of the DES-SiO_2_-MIP-QCM’s detection capability is conducted through OTC spiking. A specific concentration of OTC is introduced to the filtered water sample, and the spiked sample was analyzed using the DES-SiO_2_-MIP-QCM sensor. The results, as presented in Table 5, demonstrate standardized recoveries greater than 98%. These findings validate the effectiveness and reliability of the DES-SiO_2_-MIP-QCM sensor for OTC detection.

## 4. Conclusions

This study develops a molecularly imprinted polymer (MIP)-assisted quartz crystal microbalance (QCM) detection system to enhance the detection of oxytetracycline (OTC). The QCM detects mass changes through frequency shifts, and MIP with silica as a carrier was synthesized using diethylene glycol dimethyl ether (DES) as the monomer and SiO_2_ as the matrix, resulting in the formation of a DES-SiO_2_-MIP material. This non-toxic, cost-effective material, with nanoscale MIPs, improves OTC adsorption efficiency due to its large surface area and rapid mass transfer, achieving an adsorption capacity of 27.32 mg/g. The adsorption process follows the Langmuir model (R^2^ = 0.984), indicating well-defined binding sites for efficient OTC adsorption.

By coupling the MIP with the QCM sensor, detection performance is enhanced, providing specific mass changes due to the selective adsorption of OTC on the MIP. This enables precise concentration measurements. The sensor exhibits two linear response ranges from 0.00025~0.02 μg/mL and 0.02~1 μg/mL, and based on the low concentration linear range, obtained a detection limit as low as 0.019 ng/mL, demonstrating exceptional sensitivity. In practical applications, the DES-SiO_2_-MIP sensor successfully analyzes OTC in tap water, validating the system’s effectiveness in complex environments. This technology offers significant potential for environmental monitoring and drug residue analysis, providing reliable detection of trace OTC levels, which is crucial for water quality and drug residue management.

## Figures and Tables

**Table 1:** Roughness and thickness analysis of DES-SiO_2_-MIP in AFM results.

Sample	Rq (nm)	Ra (nm)	Vertical Distance (nm)
Bare	1.07	0.85	0.86
NIPs	17.6	14.2	33.45
MIPs	18.6	15.2	31.0
After elution	30.2	23.7	29.56

**Table 2:** The imprinting factors and selectivity factors for different pollutant.

Pollutant	Q_MIP_ (mg/g)	Q_NIP_ (mg/g)	α	β
OTC	27.23	10.03	2.71	~
DOX	14.18	9.37	1.51	1.79
CTC	11.28	7.20	1.56	1.73
TTC	13.66	9.97	1.37	1.98

**Table 3:** The selectivity factors (SF) of different pollutant detected by DES-SiO_2_-MIP-QCM and DES-SiO_2_-NIP-QCM.

	0.02 (µg/mL)	0.4 (µg/mL)	0.8 (µg/mL)	0.02 (µg/mL)	0.4 (µg/mL)	0.8 (µg/mL)
	DES-SiO_2_-MIP-QCM			DES-SiO_2_-NIP-QCM		
OTC	~	~	~	~	~	~
DOX	1.71	2.32	1.72	1.28	0.95	1.13
CTC	1.96	2.37	2.02	1.07	1.24	1.2
TTC	1.66	1.99	1.59	0.77	0.96	1.12

**Table 4:** Comparison with previous methods of OTC analysis.

Method	Linear Range	LOD	Citations
MIP-fluorescence	0.8~130 μM	0.32 μM	[54]
MIP-fluorescence	10~300 nM	8.1 nM	[55]
MIP-fluorescence	10~100 μmol/L	86 nmol/L	[56]
MIP-MEF	0.1~3.0 μM	2.40 μg/L	[57]
MIP-fluorescence	0.05~20 μg/mL	0.015 μg/mL	[58]
MIP-HPLC	10~1000 ng/mL	3 ng/mL	[59]
DES-SiO_2_-MIP-QCM	0.00025~0.02 μg/mL	0.019 ng/mL	This work

**Table 5:** Determination of OTC in real water samples.

Samples	Add (μg/mL)	Frequency Variation (Hz)	Found (μg/mL)	Recovery (%)
Tap water	0.02	−150.79	0.01963	98.15%
	0.4	−337.65	0.39530	98.83%
	0.8	−533.19	0.78843	98.55%

## Data Availability

Data will be made available on request.
